# The Efficacy of Pharmacotherapy for Decreasing the Expansion Rate of Abdominal Aortic Aneurysms: A Systematic Review and Meta-Analysis

**DOI:** 10.1371/journal.pone.0001895

**Published:** 2008-03-26

**Authors:** Idris Guessous, Daniel Periard, Diane Lorenzetti, Jacques Cornuz, William A. Ghali

**Affiliations:** 1 Department of Internal Medicine, Lausanne University Hospital, Lausanne, Switzerland; 2 Service of Angiology, Lausanne University Hospital, Lausanne, Switzerland; 3 Centre for Health and Policy Studies, Faculty of Medicine, University of Calgary, Calgary, Canada; 4 Institute of Health Economics, Edmonton, Alberta, Canada; 5 Department of Community Medicine and Public Health, Lausanne University Hospital, Lausanne, Switzerland; 6 Department of Medicine, University of Calgary, Calgary, Canada; 7 Department of Community Health Sciences, University of Calgary, Calgary, Canada; German Cochrane Center, Germany

## Abstract

**Background:**

Pharmacotherapy may represent a potential means to limit the expansion rate of abdominal aortic aneurysms (AAAs). Studies evaluating the efficacy of different pharmacological agents to slow down human AAA-expansion rates have been performed, but they have never been systematically reviewed or summarized.

**Methods and Findings:**

Two independent reviewers identified studies and selected randomized trials and prospective cohort studies comparing the growth rate of AAA in patients with pharmacotherapy vs. no pharmacotherapy. We extracted information on study interventions, baseline characteristics, methodological quality, and AAA growth rate differences (in mm/year). Fourteen prospective studies met eligibility criteria. Five cohort studies raised the possibility of benefit of beta-blockers [pooled growth rate difference: −0.62 mm/year, (95%CI, −1.00 to −0.24)], but this was not confirmed in three beta-blocker RCTs [pooled RCT growth rate difference: −0.05 mm/year (−0.16 to 0.05)]. Statins have been evaluated in two cohort studies that yield a pooled growth rate difference of −2.97 (−5.83 to −0.11). Doxycycline and roxithromycin have been evaluated in two RCTs that suggest possible benefit [pooled RCT growth rate difference: −1.32 mm/year (−2.89 to 0.25)]. Studies assessing NSAIDs, diuretics, calcium channel blockers and ACE inhibitors, meanwhile, did not find statistically significant differences.

**Conclusions:**

Beta-blockers do not appear to significantly reduce the growth rate of AAAs. Statins and other anti-inflammatory agents appear to hold promise for decreasing the expansion rate of AAA, but need further evaluation before definitive recommendations can be made.

## Introduction

Current management recommendations for patients with small abdominal aortic aneurysms (AAA) propose interval measurements of aneurysm size until elective surgical repair is indicated based on rapid expansion or size criteria (≥5.5 cm) [1]–[3]. However, AAA management based on such a “watchful-waiting” approach might not be sufficient [4]. A more proactive strategy would be to identify AAAs by screening and then to intervene therapeutically to slow down AAA expansion with preventive measures [5].

A number of pharmacotherapies have potential to limit the expansion rate of small AAAs. According to previous studies, the mean growth rate of a small AAA is 0.3–0.5 cm/year [6]. Based on this, experts propose that a reasonable therapeutic goal is to identify therapies that reduce the expansion rate from 0.5 to 0.25 cm/year (50% effectiveness) so that the typical time for a 3 cm AAA to exceed the 5.5 cm threshold for surgical consideration would be over 10 years.

According to the different AAA pathogenesis theories, a combination of biomechanical wall stress, proteolytic degradation of aortic wall connective tissue, and inflammatory/immune response may be contributing to AAA expansion over time [7]. Correspondingly, anti-inflammatory drugs (e.g., doxycycline, roxithromycine, and statins) and antihypertensive agents (e.g., ACE inhibitors, beta-blockers, diuretics, calcium antagonist) have been proposed and formally tested as pharmacological agents that may limit the expansion rate of small AAAs. Some of these agents have demonstrated an effective suppression of induced aneurysm formation in mouse models [8]–[11]. Studies evaluating the efficacy of these agents to slow down human AAA-expansion rates have also been performed [12], but they have not to date been summarized nor characterized.

Recognizing this, we performed a systematic review and meta-analysis of prospective human studies (clinical trials or cohort studies) that evaluated the efficacy or effectiveness of pharmacotherapies for reducing the expansion rate of AAA in patients with abdominal aortic diameter of 3.0 cm or greater. In conducting our review, we set out to systematically identify the full spectrum of pharmacological therapies that have been formally studied for the indication of reducing AAA expansion.

## Methods

### Search strategy

Studies were identified by searching Medline (1966 through October, 2006), EMBASE (1980 through October, 2006) and the Cochrane Controlled Clinical Trials Register (1996 through October 2006). Registered clinical trials were also searched on the www.ClinicalTrials.gov website. We limited our research to randomized controlled trials and cohort studies with a concurrent control group. We did not limit our research to any specific pharmacotherapies, nor to any limited set of languages. References of review articles and congress abstracts were also searched, and a verification Medline and EMBASE search was again performed in July 2007 to ensure that there we did not miss any newly published studies. We derived 3 comprehensive search themes that were then combined using the Boolean operator “and”. We created the first theme for AAAs by using an exploded subject heading(s) and textword terms for abdominal aortic aneurysm. The second theme for our interventions of interest was created by using the Boolean search term “or” to search for broad pharmacotherapy terms appearing as exploded subject heading(s) and textword terms. We then created the third theme for study designs of interest. Cohort studies were searched by using the terms “risk”, “prognosis”, “cohort analysis” and “follow up study” and we then used the Boolean term “or” to combine combined this with a published search filter for identifying clinical trials [13]. More information on the research strategy (i.e., subject heading(s) and textword terms) is available on request.

### Selection criteria

Two authors (IG, DP) independently reviewed each potential study for eligibility on the basis of a predefined set of eligibility criteria. AAA was defined as an aneurysm occurring below the renal arteries and with a (anteroposterior or lateral) diameter of 3 cm or more. We excluded studies that did not report original data, those assessing patients with AAAs previously treated by surgery, those concerning aneurysms of other arteries, those concerning infectious (e.g. mycotic) AAA and those with Marfan syndrome. Pharmacotherapy interventions were defined as those involving the prescription of a drug. Other interventions such as behavioural interventions (e.g. smoking cessation) were not eligible. The follow-up had to be at least 6 months and the AAA size had to be assessed on at least two occasions for studies to be included. The main outcome measure was the mean growth rate difference in AAA diameter in mm/year between pharmacotherapy and control groups and expressed with standard deviations (SD) or related measures of dispersion. To express the size of the treatment effect in each trial, we used the difference in means (MD) and the standard error of the mean difference (SEMD), which is based on the standard deviation and the number of participants for the intervention group and the control group, respectively [14]. Therefore, we included studies that either specifically reported SEMD, SD, or other measures of dispersion (i.e. 95% confidence intervals, or 25–75% interquartile ranges) from which SD could be calculated. We also included studies that did not report these estimates but that provided raw data from which SD could be calculated.

### Data extraction and quality assessment

Two individuals (IG, DP) independently extracted data from all primary studies fulfilling eligibility criteria. Abstracted information included the study design (RCT or cohort study), the characteristics of the specific pharmacotherapy assessed in each study (including dosage and duration), the participants and study characteristics (including gender, mean age, ethnicity and proportion of smokers), as well as the type of device used to measure the AAA diameter, the number of measurements performed and the proportion of patients experiencing rupture and/or undergoing surgical intervention during follow-up. We also abstracted data on other study factors that can contribute to heterogeneity in the results of efficacy of pharmacotherapy to reduce the growth rate of AAA (i.e., baseline AAA diameter, gender, age, ethnicity, smoking status, positive family history of AAA, coronary heart disease). Any discrepancies in extracted data were resolved by a third individual (WG).

The same two reviewers independently assessed the methodological quality of identified studies. The quality of included clinical trials was evaluated by using the quality assessment scale developed by Jadad and colleagues [15]. In addition to Jadad's scale, we also considered the type of allocation concealment. For cohort studies, meanwhile, we recorded the following quality indicators: the approach to participant recruitment (consecutive vs. other approaches); the length of follow-up; and the consideration of confounding factors. Finally, for both designs (RCT and cohort) we considered whether studies had been stopped early for benefit and if there was an a priori specification of sample size/power estimation.

### Statistical analysis

Studies were classified according to the type of agent used (e.g., beta-blockers) and to the study design (i.e., clinical trial vs. cohort). For pooling the results, we used the meta command in STATA. The meta command uses inverse-variance weighting to derive fixed-effects summary estimates of the treatment effect and the DerSimonian and Laird method for random estimate. To limit the sources of heterogeneity, we only estimated pooled growth rate differences for groups of studies that share the same type of agent and study design (e.g., RCTs evaluating beta-blockers). The presence of heterogeneity across trials was evaluated using a chi-square test for homogeneity [16] and random-effects and fixed-effects were used accordingly to determine pooled estimates of the growth rate difference across studies [17]. We also tested for potential publication bias using both a Begg's test for funnel plot asymmetry and an Egger's test [18], [19]. These analyses testing for publication bias were never significant across each of the meta-analyses that we performed (detailed findings are thus not reported). We conducted the statistical analyses using STATA 9.2 software (StataCorp, College Station, Texas).

## Results

**Figure 1:**
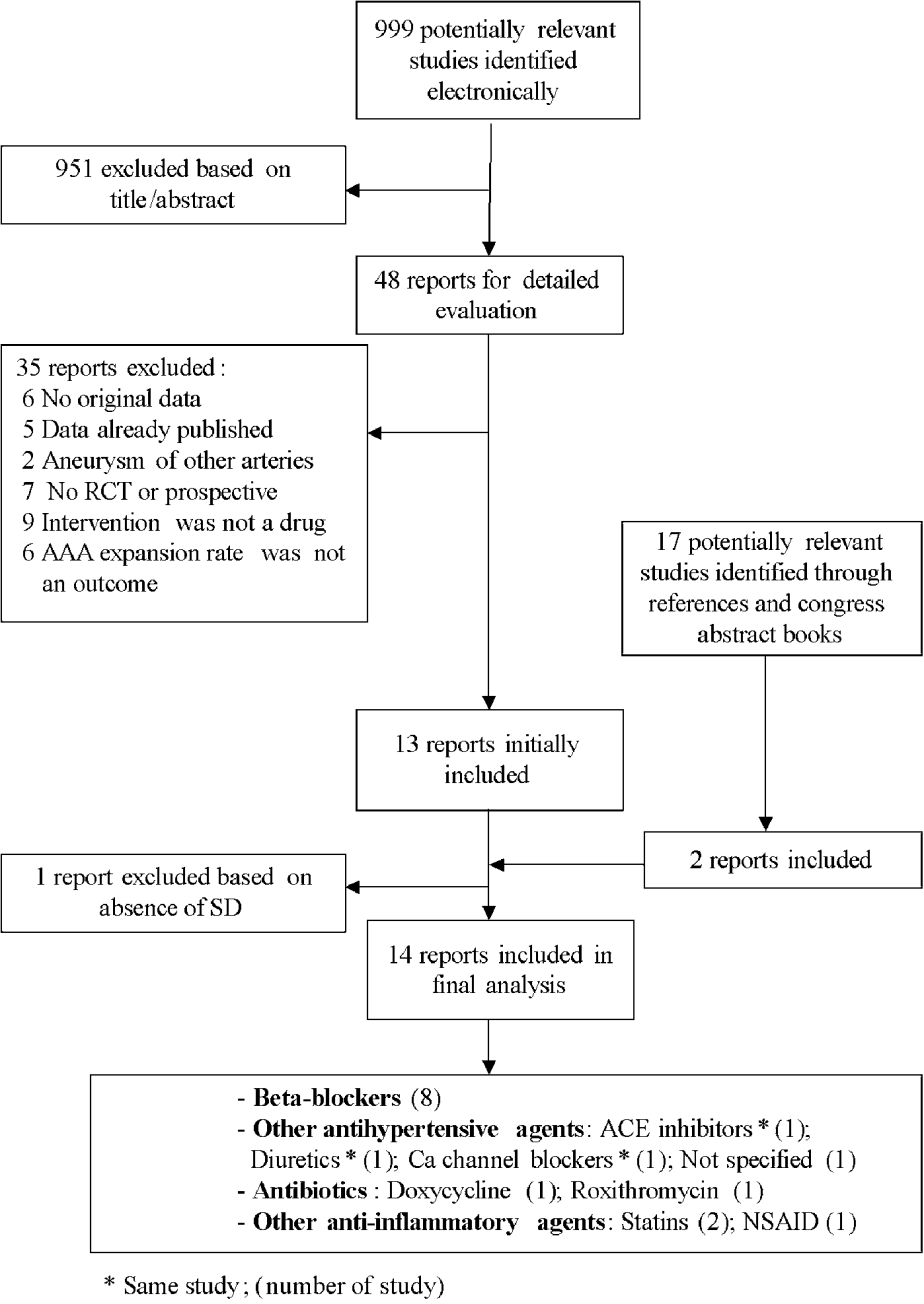
Flow chart

A total of 999 articles were identified by our initial search strategy (figure 1). After an initial screening step based on abstracts and article titles, 48 citations were judged to warrant further review (inter-observer kappa = 0.85). Among these, 35 citations were excluded for the following reasons: 6 did not report original data, 5 reported data already published, 2 concerned aneurysms other than AAAs, 7 were neither RCTs nor cohort studies, 9 were studies where the intervention of interest was not a drug, and 6 were studies where AAA expansion rate was not an outcome. We therefore identified 13 studies [20]–[32] for inclusion in our review (inter-observer kappa = 0.91 for the second step of selecting articles based on full text review). Among these selected studies, 4 studies met inclusion criteria but did not provide SD or equivalent to calculate the SEMD. We contacted the authors of these 4 studies [20], [21], [27], [32] and were successful in obtaining data needed to calculate SEMD for 3 of them [21], [27], [32].

Seventeen additional articles were then identified through a process of scanning reference lists and congress abstracts. Among these, 2 met eligibility criteria [33], [34] and were thus included in our review. As a result of this literature review process (figure 1), we identified a final total of 14 studies for inclusion [21]–[34]. Among these, one study [23] reported the results of four different pharmacological agents (table 1).

**Table 1:** Description of included studies

First Author, Year	Study design	Agent (number of participants)	Type of control (number of controls)	Dosage (SD)	Device	Mean Follow up duration in months	
						Intevention	Control
*Beta blockers*							
Lindholt [21] 1999	RCT	Propanolol (30)	Placebo (24)	40 mg/bid	US	24	24
PATI [22] 2002	RCT	Propanolol (276)	Placebo (272)	20–240 mg/d	US	30	30
Wilmink [33] 2000	RCT	Propanolol (256)	No propanolol (221)	40 mg/d	US†	34‡	33‡
Wilmink [23] 2002	Cohort†	Beta blockers* (77)	No Beta blockers (255)	NR	US	48	48
Lindholt [24] 2001	Cohort	Beta blockers* (25)	No Beta blockers (112)	NR	US	28	28
Gadowski [25] 1994	Cohort	Propanolol (21), Atenolol (10), Metoprolol (7)	No Beta blockers (83)	Propanolol 92 mg/d (38), Atenolol 68 mg/d (30), Metoprolol 80 mg/d (21)	US	43	43
Leach [26] 1988	Cohort	Propanolol (6), Selective beta blockers* (6)	No Beta blockers (15)	Propanolol 20–80 mg/d	US	27	38
Biancari [27] 2002	Cohort	Beta blockers* (17)	No Beta blockers (24)	NR	US	87	87
*Other antihypertensive agents*							
Wilmink [23] 2002	Cohort†	Diuretics* (54)	No Diuretics (278)	NR	US	48	48
Wilmink [23] 2002	Cohort†	ACE inhibitors* (24)	No ACE inhibitors (308)	NR	US	48	48
Wilmink [23] 2002	Cohort†	Ca channel blockers* (48)	No Ca channel blockers (284)	NR	US	48	48
Brady [34] 2004	Cohort	Antihypertensive* (932)	No Antihypertensive (765)	NR	US	NR	NR
*Antibiotics*							
Mosorin [28] 2001	RCT	Doxycycline (17)	Placebo (15)	150 mg/d	US	18	18
Vammen [29] 2001	RCT	Roxithromycin (40)	Placebo (44)	300 mg/d	US	18	18
*Anti-inflammatory agents*							
Schouten [30] 2006	Cohort	Simvastatin (24), Atorvastatin (19), Fluvastatin (11), Pravastatin (5)	No Statins (91)	NR	US	34	38
Sukhija [31] 2006	Cohort	Simvastatin (31), Atorvastatin (44)	No Statins (55)	20–80 mg/d	CT	23	24
Walton [32] 1999	Cohort	NSAID* (15)	No NSAID (63)	NR	US	>12	>12

* Without precision

† Same cohort study

‡ Data provided directly by the authors

NR Not reported

### Study characteristics

From 1988 to 2006, five RCTs [21], [22], [28], [29], [33] and 9 prospective cohorts studies [23]–[27], [30]–[32],[34] have evaluated the efficacy of pharmacotherapies to reduce the growth rate of AAA (table 1). A total of 4804 participants were included in these studies (1995 in intervention groups, and 2809 in control groups). Two general categories of pharmacological agents have been explored within these studies: antihypertensive agents and anti-inflammatory agents. Antihypertensive agents evaluated include beta-blockers [21]–[27], [33], diuretics [23], ACE inhibitors [23], Ca channel blockers [23] and unspecific antihypertensive agents [34]. Anti-inflammatory agents studied include antibiotics with anti-inflammatory properties (doxycycline and roxithromycin) [28], [29], statins [30], [31] and nonsteroidal anti-inflammatory drugs (NSAIDs) [32].

### Participant characteristics in included studies

Age was generally reported in the included studies (10/14) and participants' mean age was 69.0 years. Participants were mostly men in the 10 studies that reported sex of the patients [21], [22], [25], [26], [28]–[33]. The proportion of smokers (current and past smokers), an established AAA risk factor, was only available in half of the studies and varied from 17% to 72% [21], [22], [28]–[32]. The prevalence of diabetes and hypertension were only available in four studies [22], [28], [31], [32]. Although recognized as AAA risk factors, neither black race/ethnicity nor family history of AAA were reported in the included studies. Finally, among the studies reporting the proportion of participants with coronary heart disease (3/14), more than half of participants had histories of coronary heart disease [22], [30], [31].

### Annual growth rates

The mean AAA growth rates ranged across studies from −0.52 mm/year to 3.0 mm/year in the intervention groups and from 0.1 mm/year to 4.4 mm/year in the control groups. By medication category, the intervention and control AAA mean growth rates were 1.75 mm/year and 2.47 mm/year for beta blockers and controls, respectively; 1.53 mm/year and 2.87 mm/year for antibiotics and controls, respectively; and 0.74 mm/year and 3.8 mm/year for statins and controls, respectively (table 2).

**Table 2:** Annual growth rate and growth rate difference

First Author, Year	Study design	Growth rate (SD) in mm/year		Growth rate difference (mm/year)	95% CI	
		Intervention	Control			
*Beta blockers*						
Lindholt [21] 1999	RCT	3.12 (2.5)†	2.84 (2.4)†	0.28	−0.65	1.21
PATI [22] 2002	RCT	2.2 (2.9)	2.6 (3.0)	−0.40	−0.89	0.09
Wilmink [33] 2000	RCT	0.06 (0.6)	0.1 (0.6)	−0.04	−0.16	0.08
Wilmink [23] 2002	Cohort*	0.8 (2.6)	0.7 (3.2)	0.10	−0.62	0.82
Lindholt [24] 2001	Cohort	1.6 (1.2)	2.5 (2.1)	−0.90‡	−1.54	−0.26
Gadowski [25] 1994	Cohort	3.0 (3.9)	4.4 (4.2)	−1.40	−2.93	0.13
Leach [26] 1988	Cohort	1.7 (2.7)	4.4 (5.0)	−2.70	−5.69	0.29
Biancari [27] 2002	Cohort	1.56 (1.8)†	2.27 (1.9)†	−0.71‡	−1.42	0.00
*Other antihypertensive agents*						
Wilmink [23] 2002 (diuretic)	Cohort*	0.8 (2.6)	0.7 (3.4)	0.10	−0.71	0.91
Wilmink [23] 2002 (ACEI)	Cohort*	0.02 (1.6)	0.8 (2.6)	−0.78	−1.58	0.02
Wilmink [23] 2002 (Calcium blockers)	Cohort*	0.5 (2.1)	0.8 (2.5)	−0.30	−0.97	0.37
Brady [34] 2004 (antihypertensives)	Cohort	NR	NR	−0.11	−0.34	0.12
*Antibiotics*						
Mosorin [28] 2001 (doxycycline)	RCT	1.5 (2.2)	3.0 (4.3)	−1.50	−3.93	0.93
Vammen [29] 2001 (roxithromycin)	RCT	1.56 (3.6)	2.75 (4.3)	−1.19	−3.25	0.87
*Anti-inflammatory agents*						
Schouten [30] 2006 (statins)	Cohort	2.0 (1.9)	3.6 (2.9)	−1.60‡	−2.38	−0.82
Sukhija [31] 2006 (statins)	Cohort	−0.52 (3.0)	4.0 (3.0)	−4.52‡	−6.10	−2.94
Walton [32] 1999 (NSAIDS)	Cohort	2.5 (2.2)†	3.8 (2.4)†	−1.30‡	−2.59	−0.01

* Same cohort study

† Data provided directly by the authors

‡ p value<0.05

NR Not reported

### Evidence on Efficacy of Beta-blockers

Beta blockers were evaluated in 3 RCTs [21], [22], [33] and in 5 prospective cohort studies [23]–[27] and therefore are the most studied agent. One of the RCTs has only been published as an abstract [33]. A total of 1079 participants were studied in the 3 clinical trials, among whom 562 received propanolol, while 517 were control patients who did not receive beta blocker.

The other 5 cohort studies included 658 participants, among whom 169 participants were treated with beta-blockers. Propanolol was the most frequently studied agent. Its dosage ranged from 20 mg/day to 240 mg/day. For three cohorts studies, neither the type of beta-blockers nor the dosage were specifically reported [23], [24], [27]. In addition to propanolol, one cohort study also evaluated the effect of atenolol and metoprolol with mean doses of 68 mg/day and 80 mg/day, respectively [25].

**Figure 2:**
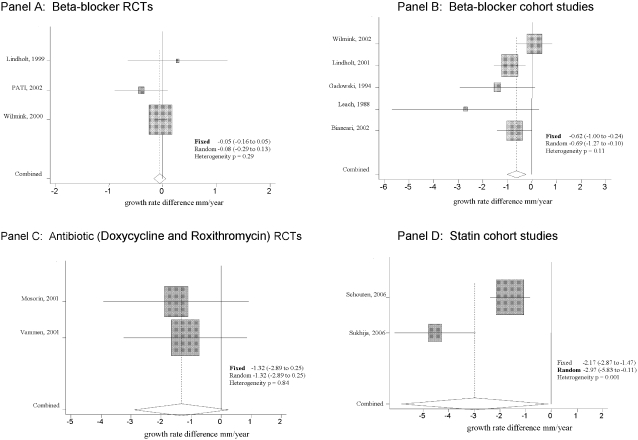
Panel A–D. Forrest plot of growth rate difference in mm/year between intervention and control group

Each of the three RCTs evaluating the efficacy of beta-blockers did not individually show a significant difference in growth rate [growth rate difference: 0.28 mm/year, (CI, −0.65 to 1.21), −0.40 mm/year, (CI, −0.89 to 0.09) and −0.04 mm/year, (CI, −0.16 to 0.08)] (table 2). Pooling of these RCTs results did not reveal a significant growth rate difference [pooled growth rate difference from a fixed effects analysis: −0.05 mm/year, (CI, −0.44 to 0.54), heterogeneity p = 0.29] (figure 2, Panel A).

Among the five beta-blockers cohort studies, two [24], [27] showed a significant growth rate reduction (table 2) and pooled results from the 5 studies combined showed a significant reduction in growth rate [pooled growth rate difference from a fixed effects analysis: −0.62 mm/year, (CI, −1.00 to −0.24), hetereogeneity p = 0.11] (figure 2, Panel B).

### Evidence on efficacy of other antihypertensive agents

In addition to beta-blockers, a cohort study by Wilmink and colleagues [23] evaluated the efficacy of three other different antihypertensive agents including diuretics, ACE inhibitors and Ca channel blockers. Brady et al. [34], meanwhile, published the results of a cohort study comparing the efficacy of ‘any antihypertensive agent’ without specifying the agents used. We report on this latter study in the general category of “other antihypertensive agents”.

Among other antihypertensive agents, all but diuretics had lower growth rates in the intervention groups relative to the corresponding control groups, but none of the observed differences were statistically significant (table 2). The growth rate differences were 0.10 mm/year for diuretics (CI −0.71 to 0.91), −0.78 mm/year for ACE inhibitors (CI −1.58 to 0.02), −0.30 mm/year for calcium channel blockers (CI −0.97 to 0.37) and −0.11 mm/year for any antihypertensive agent (CI −0.34 to 0.12) [23], [34]. Pooling of these results was not performed because of the clinical heterogeneity of the agents studied.

### Evidence on efficacy of anti-inflammatory agents (antibiotics, statins, NSAIDs)

Growth rate differences were generally larger in the studies evaluating anti-inflammatory agents than they were for anti-hypertensive agents (table 2). Growth rate difference exceeded −1.0 mm/year in favour of participants receiving doxycycline or roxithromycin and were even as large as −4.0 mm/year in one cohort study assessing statins [31]. However, upon pooling of results across studies, only statins studies reached a statistically significant level. Among the antibiotics studied, neither individual study results [growth rate difference: −1.50 mm/year, (CI, −3.93 to 0.93) and −1.19 mm/year, (CI, −3.25 to 0.87)] nor pooled results showed a significant decrease of growth rate [pooled growth rate difference from a fixed effects analysis: −1.32 mm/year, (CI, −2.89 to 0.25), heterogeneity p = 0.84] (figure 2, Panel C). In contrast, the two cohort studies assessing statins each showed a significant growth rate reduction with a corresponding pooled growth rate difference in a random effects analysis of −2.97 mm/year (95% CI, −5.83 to −0.11, heterogeneity p<0.001) (figure 2, Panel D).

Only one study by Walton et al. [32] evaluated the efficacy of NSAIDs as a therapy for reducing the AAA growth rate on the basis of its suppression of cyclooxygenase 2 activity [35]. Walton et al. reported a statistically significant median growth rate difference of −1.8 mm/year, favouring the NSAID group. We succeeded in obtaining the mean growth rate (and SD) instead of the median growth rate from the authors, and found results still favouring the NSAID group (−1.30 mm/year, 95% CI −2.59 to −0.01).

### Quality indicators for RCTs and Cohort Studies

Table 3 presents our formal assessment of the RCT quality criteria that constitute the Jadad quality assessment score. Three of the five RCTs [22], [28], [29] achieve scores of 4 or higher, and can therefore be characterized as high quality studies by that metric. Recognizing that the Jadad quality scale provides only a partial desription of RCT quality, we assessed additional RCT quality indicators and report these for each of the five RCTs idenfied in our review (table 4). It should be noted that RCT assessing beta-blockers by Wilmink and colleagues [33] is only published as an abstract; its quality was determined through review of an unpublished draft manuscript that the authors provided for our review.

**Table 3:** Jadad's score and quality indicators for RCTs

RCTs First Author, Year	Study design	Randomisation process described	Allocation sequence appropriately described	Describe as double blinding	Control treatment described as indistinguishable	Attrition described (loss of F/U, exclusion reasons)	Jadad score (0–5)
*Beta blockers*							
Lindholt [21] 1999	RCT	No	No	Yes	NO	Yes	2
PATI [22] 2002	RCT	Yes	No	Yes	Yes	Yes	4
Wilmink [33] 2000	RCT	Yes†	Yes†	No† *	No†	Yes†	3
*Antibiotics*							
Mosorin [28] 2001	RCT	Yes	Yes	Yes	Yes	Yes	5
Vammen [29] 2001	RCT	Yes	Yes	Yes	Yes	Yes	5

* Single blinded

† Data provided directly by the authors

**Table 4:** Other quality indicators for RCTs

RCTs First Author, Year	Study design	Allocation concealment described	Intention to treat analysis	Potential important baseline differences	Enough F/U duration (>12 months)	Study stopped early for benefit	Report of adverse events	Sample size pre-specified	Report of important baseline characteristics modification during F/U	Adjusted analysis for confounding variables
*Beta blockers*										
Lindholt [21] 1999	RCT	No	Yes	No	Yes	No	Yes	No	Yes	Yes
PATI [22] 2002	RCT	No	Yes	No	Yes	No	Yes	Yes	Yes	Yes
Wilmink [33] 2000	RCT	No	Yes*	No*	Yes*	No	No*	No*	No	Yes*
*Antibiotics*										
Mosorin [28] 2001	RCT	No	No	No	Yes	No	Yes	No	Yes	No
Vammen [29] 2001	RCT	Yes	Yes	No	Yes	No	Yes	Yes	No	Yes

* Data provided directly by the authors

Table 5 presents corresponding quality criteria for the 9 cohort studies that were included in our review. Of note, the most positive beta-blocker study [24] is one that fulfils only a few of the key quality criteria for cohort studies. Similarly, among the statin cohort studies, the strongly positive study by Sukhija et al. [31] also meets only a few of the measured quality criteria.

**Table 5:** Quality indicators for Cohort studies

Cohort study First Author, Year	Study design	Same mode of inclusion for intervention and control group	Enough F/U duration (>12 mionths)	Adjusted analysis for confounding variables	Mode of participants selection described	Potential important baseline differences	Report of loss of F/U	Reports of adverse events	Sample size pre-specified	Report of important baseline characteristics modification during F/U	
*Beta blockers and other antihypertensive agents*											
Wilmink [23] 2002	Cohort*	Yes	Yes	Yes	Yes	No	No	No	No		No
Lindholt [24] 2001	Cohort	No	Yes	Yes	No	No	No	No	No		No
Gadowski [25] 1994	Cohort	Yes	Yes	No	Yes	No	No	No	No		No
Leach [26] 1988	Cohort	Yes	Yes	No	Yes	No	Yes	Yes	No		No
Biancari [27] 2002	Cohort	Yes	Yes	No	Yes	No	Yes	Yes	No		No
Brady [34] 2004	Cohort	Yes	Yes	Yes	Yes	No	No	No	No		No
*Anti-inflammatory*											
Schouten [30] 2006	Cohort	Yes	Yes	Yes	Yes	Yes	Yes	No	No		No
Sukhija [31] 2006	Cohort	Yes	Yes	No	No	No	No	No	No		No
Walton [32] 1999	Cohort	Yes	Yes	No	No	No	Yes	Yes	No		No

* Same results for Wilmink 2002 Beta blockers, Ca channel blockers, diuretics and ACE inhibitors studies

## Discussion

Our systematic review reveals that a number of pharmacotherapies have been studied as potential therapies for decreasing the growth rate of AAA. In fact, by not limiting our research to a specific agent, we identified thirteen different agents that have been studied either in cohort studies or RCTs since 1988. These agents are representative of the following four classes: beta-blockers (propanolol, atenolol, metoprolol), other hypertensive agents (diuretics, ACE inhibitors, Ca channel blockers), antibiotics with anti-inflammatory properties (doxycycline, roxithromycin) and other anti-inflammatory agents (simvastatins, atorvastatin, fluvastatin, pravastatin, NSAIDs). Although the majority (11/14) of studies using these agents report a trend toward a decrease of AAA expansion, only five of them are statistically significant [23], [26], [29], [30], [31]. The pooling of observational cohort study results suggests that beta-blockers and statins may significantly reduce the growth rate of AAA.

Such a conclusion, however, is not supported by corresponding RCTs that reveal far less encouraging results for beta-blockers. The evidence on statins, meanwhile, is only from cohort studies. Observational studies are useful for assessing the relationship between exposure (e.g., pharmacotherapy) and disease (e.g., AAA), but they are subject to a number of potential biases [36]. The promising results for statins based on two observational studies are certainly important for hypothesis generation, but RCTs are now needed to confirm this hypothesis.

For years, biomechanical wall stress mechanisms, atherosclerotic processes and high blood pressure have been proposed as causes of AAA formation [37]. Accordingly, the efficacy of antihypertensive agents and particularly beta-blockers has been evaluated. Propanolol was one of the first agents considered, and although it appeared to reduce the growth rate of AAA in both animals and human, its effectiveness was limited by the fact that more than half of the patients dropped out of studies assessing this treatment because of side effects [38]. Our systematic review shows that the AAA growth rate difference between patients treated with beta-blockers vs. controls ranges across studies from −2.70 to 0.28 mm/year. However, because the encouragingly positive pooled result from cohort studies were not confirmed in RCTs, we are led to conclude that the balance of published evidence suggests that beta-blockers do *not* significantly reduce the growth rate of AAAs, and that the use of beta-blockers, and propanolol in particular, can not be recommended for this indication.

More recently, pathophysiological considerations have suggested that the progressive loss of aortic medial vascular smooth muscle cells and matrix in AAAs might be attributable to an inflammatory process due to the proteolytic depletion of medial and adventitial elastin [39], [40]. These processes involve an elastolytic proteinase called elastolytic matrix metalloproteinase (MMP). In keeping with this inflammatory hypothesis, it has also been proposed that an infectious agent, *Chlamydia pneumonia,* might be involved as an initiating or accelerating agent in the process leading to aneurysm formation and expansion [41], [42]. Accordingly, the efficacy of anti-inflammatory agents, including antibiotics, has been evaluated in a number of studies assessing the growth rate of AAAs. Antibiotics were the first anti-inflammatory agents evaluated in randomized trials. Mosorin et al. [28], assessing doxycycline, found that the overall aneurysm expansion rate during 18-month follow up was higher in the placebo group (3.0 mm) than in the doxycycline group (1.5 mm), but the difference did not reach statistical significance [28]. They attributed their lack of statistical significance to their small sample size (n = 32). Using a macrolide (roxithromycin) in a larger RCT, Vammen et al. found a significant difference between the intervention group and the control group after one year, but not after a second year of follow-up [29]. Because our analysis considered only the difference measured at the end of follow-up for all studies, our meta-analysis reports a non-sigificant finding for this latter study.

Because elastolytic matrix metalloproteinase (MMP) has been implicated in AAA formation and expansion, drugs inhibiting MMP action have been proposed as an approach to reducing the growth rate of AAAs [43]. Intriguingly, recent immunohistochemistry studies and animals studies have shown that statins possess MMP inhibiting properties [44] [Bibr pone.0001895-Nagashima1]–[Bibr pone.0001895-Luan1] [47]. In keeping with this hypothesis, the two cohort studies of statins that we identified in our review [30], [31] suggest a notable growth rate difference. As mentioned earlier, however, this evidence is from non-randomized cohort studies of statins, at least one of which [31] had suboptimal study quality according to our assessment of cohort study quality markers (table 5).

The assessment of study quality revealed that most studies reported on the key indicators of quality. The most frequently missing quality indicators concerned observational cohort studies and consisted of incomplete or lack of reporting on losses to follow-up, no report of adverse events, no sample size specification, and the suboptimal reporting of important baseline characteristics (e.g., smoking status, hypertension). Furthermore, although we carefully searched and reported on quality indicators among the included cohort studies, it should be emphasized that the most important quality indicator is actually the study design. Therefore, the results in our analysis emerging from RCTs should be considered of higher quality than results from observational cohort studies.

Our study has limitations. First, although we believe that we have identified all available studies through a comprehensive and sensitive search strategy and by seeking unpublished data from study authors, it is possible that we have missed some studies. Second, we found some evidence for statistical heterogeneity among statins studies (figure 2, panel D) and the presence of heterogeneity can compromise the interpretation [48]. These two statin studies used different classes of statins and one of the studies did not report the dose. These are clinical differences that may have contributed to the heterogeneity of results that we observed. We therefore emphasize the need to consider the corresponding pooled growth rate difference for statins with caution. A third general limitation to our review is that we would globally characterize the body of literature that we have summarized as being non-definitive, and in need of further study before firm treatment recommendations can be made. With perhaps the exception of the RCT beta-blocker evidence suggesting lack of benefit, the studies assessing other therapies (including the promising category of anti-inflammatory agents) is predominantly observational in nature, and not optimal from the standpoint of study quality and/or sample sizes. This global limitation, inherent to the body of literature that we summarized, points to a need for more RCTs assessing pharmacotherapies (especially anti-inflammatory therapies) for AAA growth rate reduction before more definitive recommendations can be made. Finally, it is worth noting that although AAA expansion rate is associated with the risk of AAA rupture [49], [50], and that expansion rate is currently used as an indication for intervention (e.g. AAA repair) [51], it is only a surrogate marker of increased rupture rate. The use of surrogate markers as endpoints presents some study feasibility advantages [52], but improvement in a surrogate endpoint does not itself confer a definitive proof of patient benefit (i.e., a decrease in risk of AAA rupture) [53]. Although both statistical and mechanistic elements suggested that AAA expansion rate is a valid surrogate marker of AAA rupture, definitive inference is only likely when using the true clinical endpoint (i.e. AAA rupture).

While we wait for such trials to be performed, clinicians will rightly pose the question of whether they should now begin prescribing various pharmacotherapies to patients with AAA? To the best of our knowledge, our study is the first to systematically review and meta-analyze the literature assessing this clinical question. Although our review findings lead us to suggest that beta-blockers do not reduce AAA growth rate, the use of surrogate markers and the small number of RCTs assessing the efficacy of beta-blockers do not definitively exclude the possibility of benefit from beta-blockers. Anti-inflammatory agents, and in particular statins, meanwhile, appear promising based on observational cohort studies. In the absence of more definitive RCT evidence, we cannot recommend use of these agents for the sole indication of reducing AAA expansion rate. If, however, clinicians are in the common scenario of having a patient with a AAA and concomitant coronary artery disease and/or hyperlipidemia, we would recommend the use of statins as these will provide established benefit for the patient's vascular disease risk, and potentially also benefit to the rate of AAA expansion.

Our review indicates that beta-blockers do not significantly reduce the growth rate of AAAs. Other pharmacotherapies, and particularly anti-inflammatory agents, hold promise for reducing the expansion rate of AAAs greater than 3cm. The literature summarized, however, constitutes a non-definitive body of evidence with most of the studies identified being observational cohort studies. There is now a need for randomized controlled trials in this area, particularly for the promising anti-inflammatory agents.
